# Fatigue Properties and Degradation of Cured Epoxy Adhesives Under Water and Air Environments

**DOI:** 10.3390/ma18174166

**Published:** 2025-09-05

**Authors:** Keiji Houjou, Haruhisa Akiyama, Kazumasa Shimamoto

**Affiliations:** Adhesion and Interface Technology Research Group, Research Institute of Core Technology for Materials Innovation, The National Institute of Advanced Industrial Science and Technology, 1-2-1, Namiki, Tsukuba 305-8564, Ibaraki, Japan; h.akiyama@aist.go.jp (H.A.); kazumasa.shimamoto@aist.go.jp (K.S.)

**Keywords:** epoxy bulk, fatigue in water, degradation, loss factor, compliance, FTIR

## Abstract

In this study, specimens cured with an epoxy adhesive were subjected to fatigue tests, which were conducted under air and water atmospheres at room temperature, because few studies have been conducted on the deformation behavior versus time (number of cycles) of the combined degradation due to moisture and cyclic stress. The epoxy adhesive was cured into plates and then cut into dumbbell-shaped specimens. Micro surface cracks were introduced into the specimen surfaces. The fatigue limit of smooth specimens without cracks in water improved compared to that in air. However, when a pre-crack was introduced at the specimen surface, all specimens fractured from the crack in water and showed the same strength as in air. Fracture toughness showed no significant difference in values between the fatigue tests in air and water. The loss factor, compliance, and creep deformation increased significantly in the fatigue tests in water compared to those for the tests in air. The specimens after testing showed that the C=O peak intensity was the same for immersion in water, fatigue in water, and fatigue in air. Therefore, no change in the chemical structure occurred during any of the loading tests.

## 1. Introduction

With the recent electrification of automobiles and automated driving, vehicle weight has increased significantly owing to the addition of batteries, sensors, and other components. Consequently, there is an urgent need to reduce the weight of vehicle bodies. Multi-material bonding is the best way to reduce weight, increasing the need for adhesive bonding. However, adhesive bonding is less reliable than mechanical fastening, with extensive research devoted to mechanical evaluation [1,2,3,4].

The most serious problem is adhesive deterioration owing to moisture absorption. Therefore, extensive research has been conducted on deterioration due to moisture and high temperatures [5,6,7,8,9]. To understand the degradation characteristics of adhesive joints, it is essential to study the degradation characteristics of adhesives. Similarly, to comprehensively understand the degradation properties of adhesive joints, it is necessary to study the degradation of cured adhesives. Accordingly, the mechanical properties and durability of adhesively cured specimens (hereafter referred to as bulk specimens) have been studied in detail [10,11,12,13].

In a previous study, the effect of cyclic stress in air at room temperature on the fatigue and creep properties of cured adhesives was investigated [13]. However, structural adhesive joints are subjected to stress while being exposed to rainwater in service. Previous studies have investigated the degradation due to moisture and fatigue [14,15,16,17,18,19,20,21]. However, few studies have been conducted on the deformation behavior versus time (number of cycles) of the combined degradation due to moisture and cyclic stress [19,21]. Rodriguez-Copio et al. [19] reported an increase in specimen absorption when bulk epoxy was immersed in water under stress, but the applied stress was static. For practical applications, water absorption data under repeated strain is required. Savvilotidou et al. [21] reported on the mechanical properties per cycle number, which were fatigue tested under wet and dry conditions, focusing mainly on Young’s modulus and strength, without evaluating fracture mechanics.

Based on this background, in this study, bulk fatigue tests were conducted in water to clarify differences in degradation behavior compared with air. It would be of interest to investigate whether the combined effects of cyclic stress and moisture absorption break the molecular bonds and cross-links in polymers.

One of the authors of this paper studied the effects of cracks on the surface of metal specimens [22,23,24,25]. Cracks become harmless owing to the residual stress caused by shot peening. However, polymers absorb water and consequently become soft. Therefore, microcracks are expected to be harmless because the crack tip is insensitive to stress. To clarify the fracture mechanical behavior of specimens in water and air, fatigue tests were conducted by introducing microcracks on the specimen surface.

The purpose of this study is to clarify differences in fatigue degradation behavior in water compared with air through bulk fatigue testing.

The remainder of this paper is organized as follows. Section 2 covers the test specimens and experimental methods used. Section 3 describes the differences in fatigue strength and deformation behavior between air and water environments, along with the chemical structural changes caused by strain. Finally, Section 4 summarizes the conclusions.

## 2. Materials and Methods

### 2.1. Adhesive Material

In this study, an epoxy thermosetting adhesive (Cemedine Co., Ltd., Koga, Japan), which is commonly used in automobiles, was selected as the adhesive. Its chemical composition is presented in Table 1. Carboxyl-terminated butadiene acrylonitrile (CTBN) rubber was added to improve the adhesive’s elongation and toughness. The mechanical properties of the cured adhesive are shown in the lower rows of Table 1. The main component is epoxy resin, with dicyandiamide as the hardener, resulting in a sequential polymerization mechanism. Furthermore, thermal curing was performed under conditions where no residual epoxy groups were detected by IR, indicating that monomer residue is virtually absent.

### 2.2. Specimens

**Figure 1:**
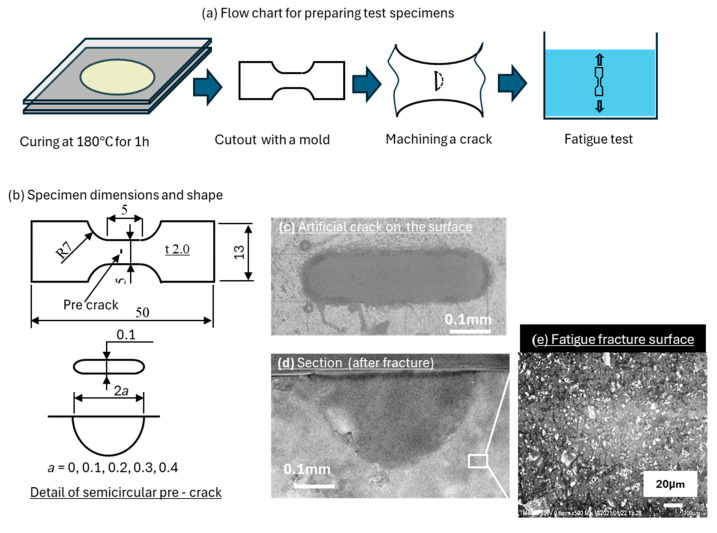
Dimensions and shapes of the specimens tested in this study. (**a**) Flow chart for test specimen preparation. (**b**) Dimensions of the dumbbell-shaped specimen. (**c**) Image of the artificial crack on the specimen surface. (**d**) Image of the crack surface after fracture. (**e**) SEM image of the fracture surface in detail.

Figure 1 shows the specimen preparation process, specimen shape, and a detailed view of the artificial crack. The adhesive was sandwiched between two glass plates, controlled to a thickness of 2 mm, and cured at 180 °C for 1 h in an electric furnace. Then, a dumbbell-shaped specimen, as shown in Figure 1b, was cut from the adhesive-cured plate. The specimen shape does not conform to a standard; its dimensions were determined by the apparatus and jig used for testing in water. Subsequently, semicircular artificial cracks with radii of *a* = 0.1, 0.2, 0.3, and 0.4 mm and a width of 0.1 mm were introduced on the central surface using a numerically controlled machine tool with a ball end mill. The surface cracks were machined perpendicular to the stress direction. The pre-crack shapes were confirmed by observing the fracture surfaces. Figure 1c,d show photographs of the surface and cross section of a pre-crack with *a* = 0.2 mm. Figure 1e shows an SEM image of the fracture surface in the air test. White CaCO_3_ (filler) particles are visible in the epoxy matrix, while the other composite materials were difficult to identify.

### 2.3. Experimental Procedure

A fatigue tester (EHF-LV010KN Servo Pulser, Shimadzu Corporation, Kyoto, Japan) was used in this study. The frequency and stress ratio were load-controlled sine waves with *f* = 10 Hz and *R* = 0, respectively. The accompanying Shimadzu software 4830 recorded load and displacement waveforms, from which the storage modulus *E*′, loss modulus *E*″, and loss factor tan *δ* = *E*″/*E*′ were calculated.

**Figure 2:**
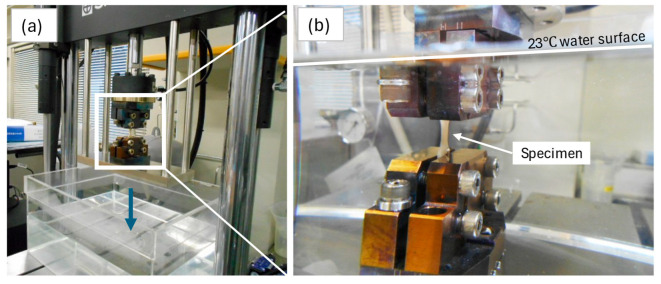
Images of the fatigue test (**a**) equipment and (**b**) aquarium.

Figure 2 shows images of the fatigue test conducted in water. Figure 2a,b show the fatigue test machine and testing in water, respectively. Purified water was used to prevent impurities from affecting the specimens. The displacements and loads were monitored during the test, and the loss factor and compliance were measured. A test in which the number of cycles would not fracture up to 2 × 10^6^ times was terminated, and the corresponding stress was defined as the fatigue limit *σ*_wa_. The fracture surfaces were observed with an optical inverted microscope BX53M (OLYMPUS Corp., Hachioji City, Japan), and the fatigue crack sizes were measured. Fourier-transform infrared spectroscopy (FTIR; 4300FTIR; Agilent Corp., Santa Clara, CA, USA) measurements were performed to study the effects of stress and moisture on the chemical structure of the specimens.

## 3. Results and Discussion

### 3.1. Fatigue Tests for Smooth Specimens in Water and Air

Adhesive joints used outdoors are exposed to moisture and stress. Therefore, the fatigue tests were performed on the specimens in water and air at room temperature (23~25 °C).

**Figure 3:**
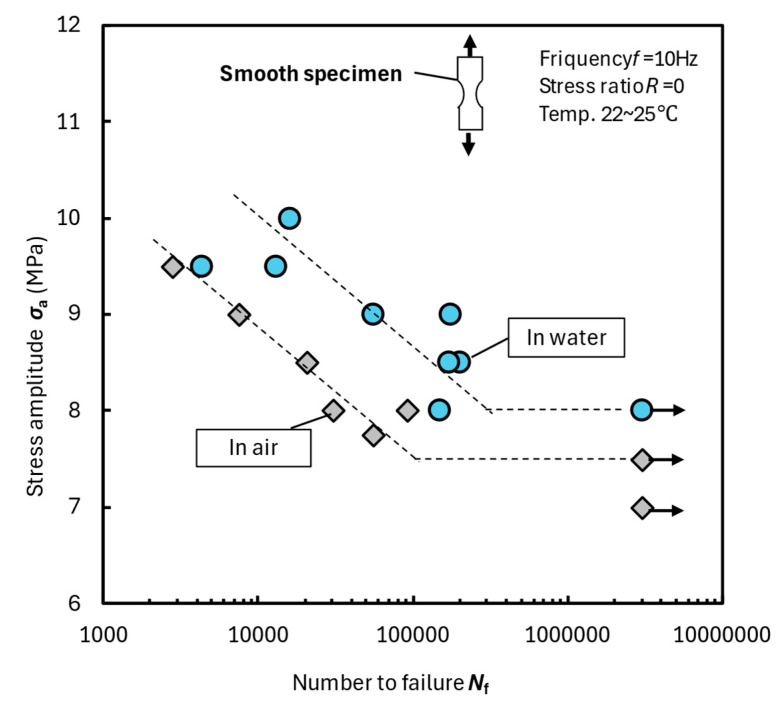
Fatigue test results for smooth bulk specimens in water and air at 23 °C.

Figure 3 shows the fatigue test results for smooth specimens. The arrows in the figure indicate the test termination. The fatigue strength (stress amplitude *σ*_a_) in water was higher than that in air at all stress levels and exhibited a wide range of values. Inflection points in the fatigue curves appeared between the numbers to failure (*N*_f_) of 100,000 and 200,000. Therefore, the maximum stress amplitude that did not cause fracture up to 2–3 × 10^6^ cycles was defined as the fatigue limit *σ*_wa_, as mentioned above. The fatigue limits in air and water were *σ*_wa_ = 7.5 and 8 MPa, respectively. The smooth specimens exhibited a higher fatigue strength in water than in air.

As will be discussed later, when an epoxy-based specimen absorbs moisture, it softens and elongates. Subsequently, microcrack formation on the surface was prevented, and, consequently, the fatigue strength increased. Fatigue normally occurs when stress concentration generates microcracks localized in small areas that subsequently grow. However, when polymers absorb water, the increased elongation is expected to release stress concentration.

### 3.2. Fatigue Tests for Bulk Specimens with a Surface Microcrack in Water

The fatigue test results for smooth specimens exhibited an improvement in strength in water. Therefore, the effect of surface microcracks is expected to be reduced. Bulk specimens with surface cracks were subjected to fatigue testing in water, and the results were compared to those in air.

**Figure 4:**
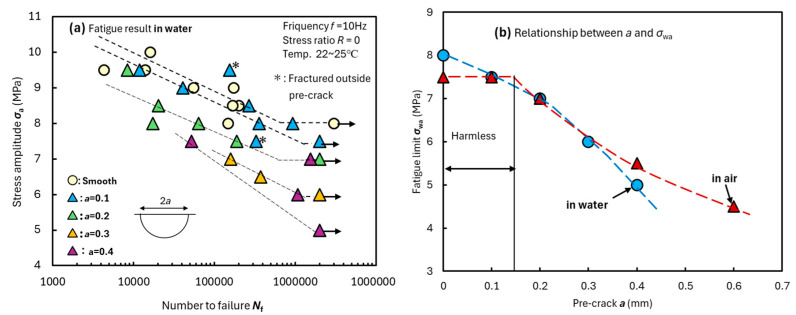
(**a**) Fatigue test results for pre-cracked bulk specimens in water at 23 °C. (**b**) Relationship between pre-crack size *a* and fatigue limit *σ*_wa_ [13].

The fatigue test results for bulk specimens in water are shown in Figure 4a. The frequency and stress ratio were set as *f* = 10 Hz and *R* = 0, respectively. For the fatigue tests of cracked specimens, eight test pieces were prepared for *a* = 0–0.2, and four test pieces were prepared for the other crack sizes. However, specimens that fractured due to large internal defects were excluded. The fatigue limit decreased as the pre-crack size increased. The asterisk (*) in the figure indicates a test that fractured outside the pre-crack. In the fatigue tests in air, no specimen fractured from the pre-crack with *a* = 0.1 mm, and the fatigue strength was equivalent to that of smooth specimens [13]. In previous studies, a “harmless crack” was defined as a crack in which the fracture originated outside the pre-crack and the fatigue limit was equal to or higher than that of a smooth specimen [22,23,24,25]. In this study, pre-cracks with *a* = 0.1 mm were considered harmless in the fatigue tests in air. However, in the test in water, the fatigue limits of the pre-crack specimens were the same as those of smooth specimens; however, almost all specimens failed from the pre-crack at all stress levels. Therefore, all pre-cracks, including those with *a* = 0.1, were not harmless and affected the fatigue failure. The specimen’s surface became insensitive to moisture, making it difficult for stress concentration to occur. The fracture origins were concentrated at the pre-cracks because they were the only stress-concentration areas.

The relationship between pre-crack size *a* and fatigue limit *σ*_wa_ is shown in Figure 4b. As shown in the test results in air (indicated by the △ symbols), the maximum harmless crack size is located between pre-cracks with *a* = 0.1 and 0.2 mm. However, the test results in water (indicated by the ○ symbols) did not exhibit harmless cracks. Both strengths of cracked specimens over *a* = 0.1 mm were equivalent, because the pre-cracks were the primary failure factor in both environments. In crack-free specimens tested underwater, swelling delayed crack initiation. However, for surface pre-cracks deeper than 0.1 mm, stress was selectively concentrated, leading to fracture from the pre-crack.

**Figure 5:**
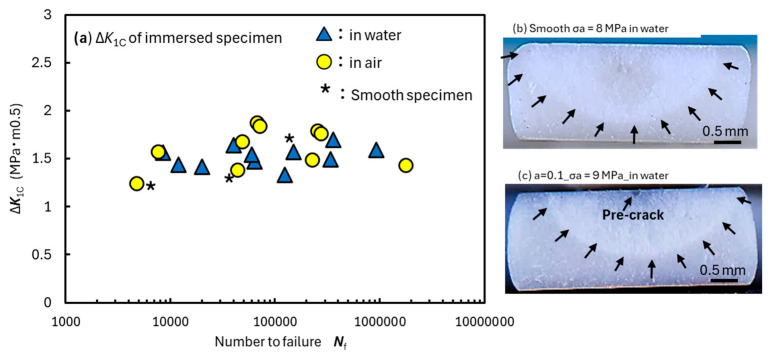
(**a**) Effect of *N*_f_, pre-crack, and immersion on Δ*K*_1C_. Images of the fracture surface for (**b**) smooth and (**c**) pre-cracked specimens for *σ*_a_ = 8 MPa in water [13].

The Newman–Raju solution was then used to calculate the fracture toughness Δ*K*_1C_ from fracture surface observations. It is worth noting that out-of-spec sizes and corner cracks were excluded because they were not applicable to the calculations [26]. Figure 5a shows the relationship between the number to failure *N*_f_ and Δ*K*_1C_. The semi-elliptical cracks, which are indicated by arrows in Figure 5b,c, are fatigue cracks formed during the fatigue test in water. This crack size and maximum applied stress were used to calculate Δ*K*_1C_. The asterisk (*) in the figure represents the test results for the smooth specimen (*a* = 0). Δ*K*_1C_ ranged between 1.3 and 1.8 MPa·m^0.5^ for all tests and was not affected by *N*_f_, *σ*_a_, *a*, and moisture absorption. The symbol (▲*) in Figure 5a represents the test result when a smooth specimen (without pre-crack) fractured in water. Even under water fatigue testing, the fracture origin was always located on the surface. In other words, the improvement in fatigue strength in water observed in Figure 3 is due to the delay in crack initiation caused by swelling and softening.

### 3.3. Fatigue and Deformation Behavior of Specimens

In the fatigue experiments, the bulk specimens deformed over time. Bulk displacements were extracted from the results, and the deformation behavior was analyzed. The differences in the deformation behavior in air and water were also investigated, as discussed in Section 3.4.

#### 3.3.1. Compliance Behavior Transition in the Fatigue Tests

**Figure 6:**
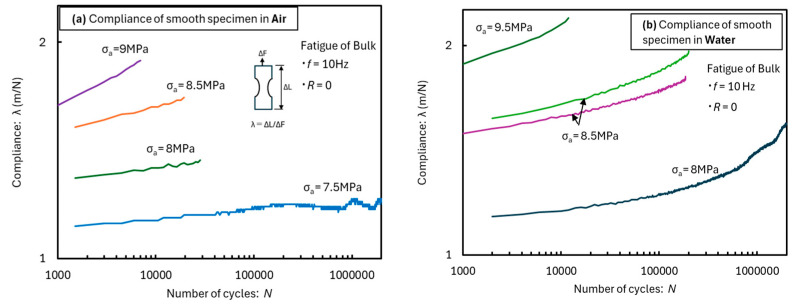
Compliance behavior transition for smooth bulk specimens in the fatigue test in (**a**) air and (**b**) water.

The relationship between the cyclic stress number and compliance *λ* is shown in Figure 6. Owing to the long test time, it was difficult to take digital image correlation (DIC) photographs. Therefore, a relative evaluation was conducted by obtaining *λ* from the specimen displacement instead of Young’s modulus. Compliance λ represents the elongation relative to the load and depends strongly on the Young’s modulus of the material. *λ* was obtained as follows: *λ* = Δ*L*/Δ*F*, where Δ*L* is the elongation range of the entire specimen and Δ*F* is the load range. Figure 6a shows the test results in air. As can be seen, *λ* exhibits a good linear relationship with the power function of the number of cycles *N* and increases as the applied stress increases. However, when the stress (*σ*_a_ = 7.5 MPa) that showed the fatigue limit was applied, *λ* did not increase until the end of the test. Figure 6b shows the test results in water. As can be seen, *λ* does not exhibit a linear relationship with *N*. In the case with *σ*_a_ = 8 MPa, which showed a fatigue limit, *λ* continued increasing until the end of the test. This is the main reason for the free volume increase due to absorbed moisture, influencing *λ*.

#### 3.3.2. Creep Deformation Transition in the Fatigue Tests

**Figure 7:**
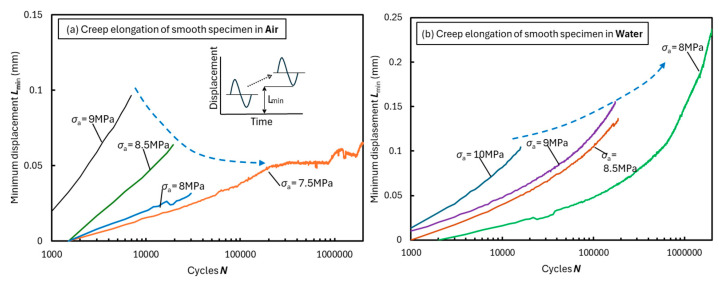
Creep elongation behavior transition for smooth bulk specimens in the fatigue test in (**a**) air and (**b**) water.

Creep deformation was compared between the fatigue tests in air and water. In the fatigue tests for bulk specimens, deformation owing to crack growth and creep elongation progresses simultaneously [13]. The increase in displacement amplitude during fatigue testing can be regarded as crack growth. However, the increase in the lower limit of the amplitude *L*_min_ is regarded as creep deformation, as shown in Figure 7. Figure 7a shows the fatigue test results in air. If the cyclic stress increases, the life increases and deformation decreases, as indicated by the dashed arrows. Meanwhile, Figure 7b shows the fatigue test results in water. As the cyclic stress increased, as indicated by the dashed arrows, both the life and deformation increased. The allowable elongation increases with time in a water-absorbing environment. Therefore, under high stress, the material fractured before sufficient moisture was absorbed. However, under low stress, both the elongation and lifetime improved because there was sufficient time to absorb moisture for elongation.

#### 3.3.3. Transition of the Viscoelasticity Behavior

**Figure 8:**
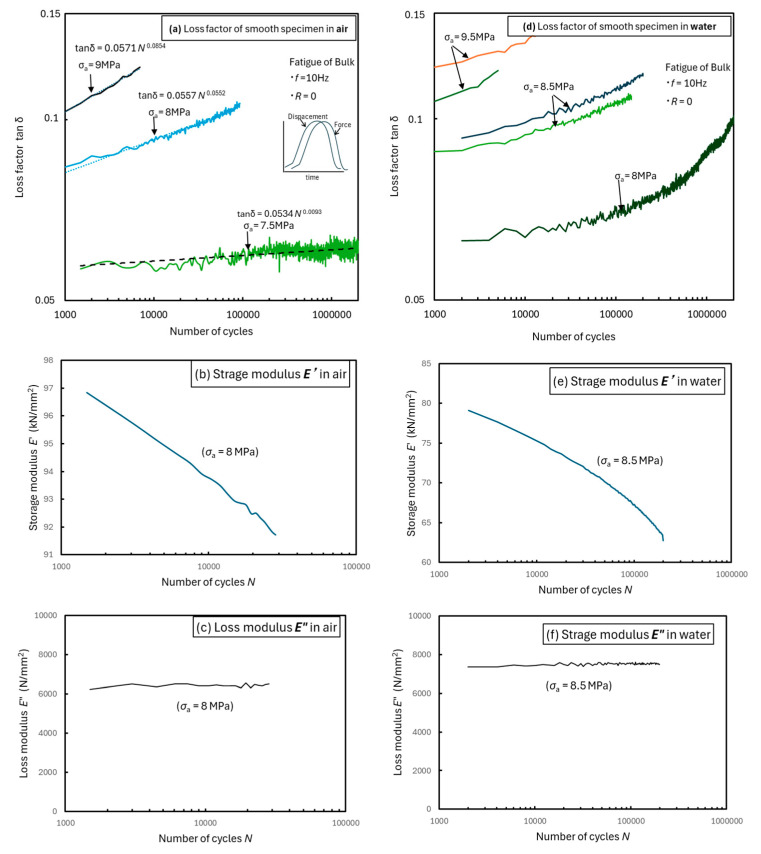
Behavior of loss factor, storage modulus, and loss modulus for smooth specimens in the fatigue tests in (**a**–**c**) air and (**d**–**f**) water.

Because the epoxy bulk is a viscoelastic material, a phase difference occurs between the displacement and load waveforms. The degree of degradation can be determined from phase differences. The relationship between the number of cycles and loss factor tan *δ* is shown in Figure 8. tan *δ* was determined using Equations (1)–(4).

(1) Loss angle: δ=2π∆t·f,

where Δ*t* is the phase difference between stress and strain, and *f* is the frequency.

(2) $$\mathrm{Storage}\;\mathrm{modulus}:\;E^{’}=\left|E\right|\cos\delta ,$$

(3) $$\mathrm{Loss}\;\mathrm{modulus}:\;E\prime \prime =\left|E\right|\sin\delta ,$$

(4) $$\mathrm{Loss}\;\mathrm{factor}:\;\tan\delta =\frac{E^{\prime \prime }}{E^{’}}$$

Figure 8a shows the test results in air. Tan *δ* exhibits a good linear relationship with the power function of *N* and shows almost no increase below the fatigue limit. Comparing the results shown in Figure 6a, Figure 7a, and Figure 8a, it can be seen that the compliance, creep deformation, and loss factor hardly changed under the fatigue limit stress. Therefore, the integrity of a polymer is maintained if the service stress is designed to be below the fatigue limit *σ*_aw_, which is an extremely important parameter. Figure 8b shows the test results in water. Although these results exhibit fatigue limits (*σ*_a_ = 8 MPa), tan *δ* increased until the end of the test. In particular, the slope on the high-frequency side increases rapidly. These results are similar to those of the compliance analysis.

As shown in Figure 8a,d, tan *δ* was small (<<1) in both air and water, exhibiting behavior similar to that of solids.

Furthermore, energy dissipation due to viscosity (*E″*) remains constant in both air and water (Figure 8c,f). However, the storage modulus (*E′*) decreases due to relaxation and dissociation of the cross-linked structure caused by fatigue.

Furthermore, it was also shown that structural degradation does not occur at the fatigue limit in air. It was concluded that fatigue in this material initiates with relaxation and disintegration of the cross-linked structure.

### 3.4. Deformation Behavior Under Static and Cyclic Stress in Water

Fatigue testing of the bulk specimens in water was found to increase compliance, tan *δ*, and creep deformation compared with the results in air, and the reasons for these differences were investigated.

**Figure 9:**
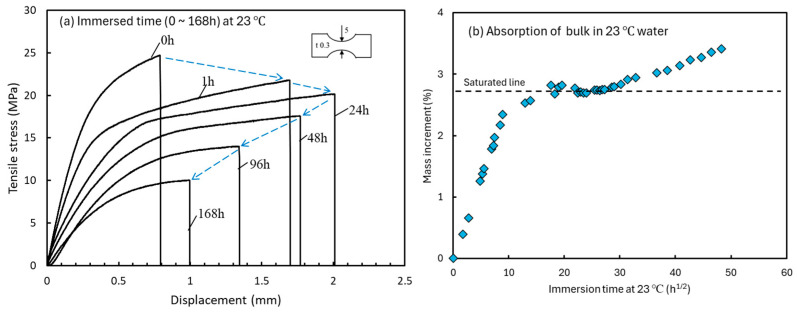
Moisture absorption properties of epoxy bulk specimens. (**a**) Relationship between immersion time and the stress-strain curves. (**b**) Relationship between immersion time at 23 °C and the percentage of water absorption [12].

Figure 9a shows the relationship between immersion time and the stress-displacement curves [27]. However, the horizontal axis was modified from [27] and expressed as strain ε. The ε represents the displacement divided by the length of the parallel section as strain. Also, the specimen thickness was 0.3 mm. Assuming that the moisture diffusion into the bulk specimens follows Fick’s law, the water absorption rate was approximately 44 times that of the present specimen (*t* = 2 mm). The diffusion coefficient calculated from the water absorption ratio up to 20 h^1/2^ after immersion was 8.68 × 10^−4^ mm^2^/h [12]. The fatigue tests conducted in this study correspond to the behavior shown in Figure 9a before 24 h of immersion. Therefore, elongation continued increasing during the whole fatigue test. Because the slope of the straight line (Young’s modulus) in Figure 9a decreases with immersion time, the compliance in Figure 6 increases as the number of cycles (time) increases. Figure 9b shows the relationship between immersion time at 23 °C and the percentage of water absorbed in the bulk specimens [12]. The two-stage increase in water absorption is attributed to changes in molecular bonds during long-term immersion, as indicated by FTIR measurements, resulting in an increase in the saturated water absorption capacity [28,29,30]. Considering the specimen thickness, in this study, the fatigue limit corresponded to that before the saturation line in Figure 9b. Therefore, the specimen elongation results shown in Figure 6b and Figure 7b are in the initial monotonic absorption range. Figure 9b shows that the maximum water absorption rate of the test material is 2.7%, which is relatively high. Water absorption decreases cross-link density and loosens the structure, resulting in softening and increased elongation. This, in turn, improves the fatigue strength in water, as shown in Figure 3.

**Figure 10:**
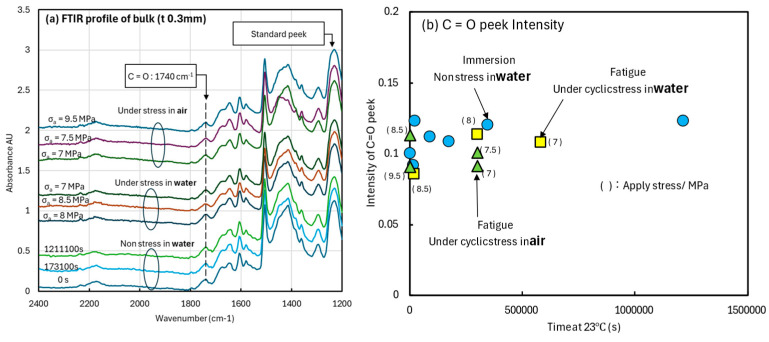
FTIR measurement results. (**a**) Normalized profiles of the tested specimens. (**b**) Relationship between tested time and intensity of the C=O peaks.

To clarify these deformation behaviors, the effects of cyclic stress and moisture on the chemical bonding in the bulk specimens were investigated. The relationship between the moisture and stress-loading conditions and the FTIR profiles was studied. Figure 10a shows the FTIR profiles measured on the surface of the specimens subjected to the initial conditions, non-stress immersion, and fatigue testing in air and water. The measurement points were located near the fracture surface. The profiles were subtracted with the 1800-cm^−1^ peak intensity as the background and normalized by the 1237-cm^−1^ peak in the fingerprint area as the standard intensity. The peak intensity at 1740 cm^−1^, indicated by the vertical dashed line, represents the C=O bond [31,32], which indicates molecular bond disconnection, oxidation, and degradation progression. This is because, under high temperature and high humidity, the 1650 cm^−1^ C=C bond and 2170 cm^−1^ C≡N bond peaks decrease, while the 1740 cm^−1^ C=O bond peak increases, suggesting oxidation of the adhesive [27]. Furthermore, previous studies have investigated the degradation mechanism of this adhesive and found that the strength of the 1740 cm^−1^ peak strongly correlates with adhesive strength [33]. The lower group in Figure 10a represents profiles immersed in water at 23 °C for up to 1.2 × 10^5^ s. The middle group represents the profiles of the specimens fatigue tested in water at 23 °C, and the upper group represents the profiles of the specimens after fatigue testing in air at 23 °C. The stress amplitudes are indicated by the profile lines. The C=O peak (1740 cm^−1^) intensities are summarized in Figure 10b. The horizontal axis represents the test time calculated from the number of cycles. The C=O peak intensity remained unchanged in both fatigue tests (air and water) and immersion tests. In other words, although high temperatures can alter chemical bonds [13], cyclic strain in room-temperature water did not change the chemical bonds or structure.

The increase in specimen compliance, elongation, and tan *δ* with cyclic strain is due to the swelling, free volume, and range of motion increase because of water absorption.

## 4. Conclusions

Bulk specimens were prepared from an epoxy adhesive, and fatigue tests were performed in room-temperature water and air. Based on the results, the main conclusions of this study can be summarized as follows:

- Fatigue strength and limit values in water were improved by 7% compared to those in air.
- In fatigue tests conducted in water on specimens with artificial surface microcracks, all fractures originated from existing cracks. Therefore, pre-cracks of 0.1 mm or larger were found to affect fracture behavior in water.
- The fracture toughness value (1.5 MPa·m^0.5^) calculated from the tests in water was the same as that in air, indicating that water absorption had no effect on the toughness of pre-cracks.
- The compliance, creep deformation, and tan *δ* were higher in fatigue tests in water compared to those in air. The deformation was mainly due to expansion caused by water absorption.
- The FTIR measurement results showed that chemical structural transition did not occur regardless of load, time, or absorption.

## Figures and Tables

**Table 1:** Chemical composition and mechanical properties of the adhesive.

Material	Mass%
Bisphenol A epoxy resin	24
CTBN-modified epoxy resin (elastomer 40%) CTBN; carboxyl-terminated butadiene acrylonitrile rubber	39
Fumed silica	3
Filler (CaCO_3_)	26
CaO	2
Dicyane diamide	5
3-(3,4-dichlorophenyl)-1,1′-dimethylurea	1
**Bulk Mechanical Properties**	
Tensile strength at R.T. (MPa)	30
Young’s modulus at R.T. (MPa)	1100
Poisson’s ratio at R.T.	0.41
*T*_g_ point (°C)	110–125

## Data Availability

The original contributions presented in this study are included in the article. Further inquiries can be directed to the corresponding author.
